# Myofibroblast transdifferentiation is associated with changes in cellular and extracellular vesicle miRNA abundance

**DOI:** 10.1371/journal.pone.0256812

**Published:** 2021-11-11

**Authors:** Siti Amalina Inche Zainal Abidin, Ian Charles Paterson, Stuart Hunt, Daniel W. Lambert, Samuel Higginbotham, Ryan Charles Pink

**Affiliations:** 1 Department of Oral and Craniofacial Sciences, Faculty of Dentistry, University of Malaya, Kuala Lumpur, Malaysia; 2 Oral Cancer Research & Coordinating Center, Faculty of Dentistry, University of Malaya, Kuala Lumpur, Malaysia; 3 Integrated Biosciences, School of Clinical Dentistry, University of Sheffield, Sheffield, United Kingdom; 4 Kroto Research Institute, University of Sheffield, Sheffield, United Kingdom; 5 Department of Biological and Medical Science, Faculty of Health and Life Sciences, Oxford Brookes University, Oxford, United Kingdom; Universitat des Saarlandes, GERMANY

## Abstract

Transforming growth factor-beta 1 (TGF-β1), a pro-fibrotic tumour-derived factor promotes fibroblast differentiation in the tumour microenvironment and is thought to contribute to the development of pro-tumourigenic cancer-associated fibroblasts (CAFs) by promoting myofibroblast differentiation. miRNA dysregulation has been demonstrated in myofibroblast transdifferentiation and CAF activation, however, their expression varies among cell types and with the method of fibroblast induction. Here, the expression profile of miRNA in human primary oral fibroblasts treated with TGF-β1, to derive a myofibroblastic, CAF-like phenotype, was determined compared to untreated fibroblasts. Myofibroblast transdifferentiation was determined by the expression of alpha-smooth muscle actin (α-SMA) and fibronectin-1 extra domain A (FN-EDA1) using quantitative real-time PCR (qRT-PCR) and western blot. The formation of stress fibres was assessed by fluorescence microscopy, and associated changes in contractility were assessed using collagen contraction assays. Extracellular vesicles (EVs) were purified by using size exclusion chromatography and ultracentrifugation and their size and concentration were determined by nanoparticle tracking analysis. miRNA expression profiling in oral fibroblasts treated with TGF-β1 and their extracellular vesicles was carried out using tiling low-density array cards. The Database for Annotation, Visualization, and Integrated Discovery (DAVID) was used to perform functional and pathway enrichment analysis of target genes. In this study, TGF-β1 induced a myofibroblastic phenotype in normal oral fibroblasts as assessed by expression of molecular markers, the formation of stress fibres and increased contractility. TaqMan Low-Density Array (TLDA) analysis demonstrated that miR-503 and miR-708 were significantly upregulated, while miR-1276 was significantly downregulated in TGF-β1-treated oral fibroblasts (henceforth termed experimentally-derived CAF, eCAF). The gene functional enrichment analysis showed that the candidate miRNAs have the potential to modulate various pathways; including the Ras associated protein 1 (Rap1), PI3K-Akt, and tumour necrosis factor (TNF) signalling pathways. In addition, altered levels of several miRNAs were detected in eCAF EV, including miR-142 and miR-222. No differences in size or abundance of EV were detected between eCAF and normal oral fibroblast (NOF). Little overlap was observed between changes in cellular and EV miRNA profiles, suggesting the possibility of selective loading of EV miRNA. The study reveals miRNA expression signature could be involved in myofibroblast transdifferentiation and the miRNA cargo of their EV, providing novel insight into the involvement of miRNA in CAF development and function.

## Introduction

Several lines of evidence have demonstrated that the altered stroma microenvironment makes a significant contribution to the malignant progression of cancers, including oral squamous cell carcinomas (OSCC) [1]. The tumour microenvironment is a dynamic milieu consisting of a mixture of cancerous cells together with stromal cell populations. The majority of tumour stromal cells are activated fibroblasts which commonly express α-SMA [2] and FN-EDA1 [3]. TGF-β1 is a well-established factor capable of inducing myofibroblast phenotype *in vitro* [4]. Activin A, a member of the TGF-β family of proteins, provokes myofibroblast differentiation, similar to that of fibroblasts stimulated with TGF-β1 [5]. Other growth factors and cytokines mediate myofibroblast transdifferentiation including interleukin-6 (IL-6), platelet-derived growth factor (PDGF) and connective tissue growth factor (CTGF) [6]. Taken together, various cytokines and growth factors are capable of modulating the myofibroblast transdifferentiation, although the precise signals, and underlying molecular mechanisms, promoting the formation of myofibroblastic CAF *in vivo* remain to be fully elucidated.

miRNAs are small non-coding RNA molecules that regulate target gene expression and modulate various biological processes [7]. Dysregulation of miRNA expression has been implicated in myofibroblast transdifferentiation and the CAF phenotype [3, 8–10]. Shen *et al* [11] reported the upregulation of miR-7 in CAFs of oral cancer compared with their paired normal fibroblasts, and inhibition of miR-7 induces a functional change of CAFs into normal fibroblasts. Thus, differently expressed miRNAs in myofibroblasts opens the possibility of a treatment approach targeting the tumour stroma with miRNA modulators (mimic or inhibitor). In addition to their cell-autonomous effects, miRNAs are increasingly recognised to play a role in intercellular signalling via their presence in EV. EV, encompassing exosomes, microvesicles and other nano-scale lipid encapsulated vesicles, are released by every cell type and present in every body fluid studied to date. They carry 2cargo including RNA, DNA, protein and lipid, and have been implicated by a range of evidence to induce responses in recipient cells, at least in part by the transfer of functional miRNA. Although the miRNA cargo of EV derived from cancer cells has been widely studied, much less is known about their presence or functional roles in fibroblast-derived EV.

While most studies focused on the effects of miRNA-derived CAFs on cancer progression, only limited information is available regarding the functional role of miRNA in regulating myofibroblast transdifferentiation. In this study, we have successfully characterised a myofibroblastic, CAFs-like phenotype in oral fibroblasts. We have further determined the differential miRNA expression; miR-503 and miR-708 were upregulated, while miR-1276 was downregulated in eCAFs. Furthermore, we identified pathways potentially regulated by altered miRNA that may influence CAF phenotype and function. In addition, we demonstrate the presence of a wide array of miRNA in fibroblast and eCAF-derived EV and identify differences in EV cargo that may potentially contribute to their pro-tumorigenic functions.

## Materials and methods

### Cell culture

Fibroblast cultures (NOF804 and NOF822) were kindly provided by Dr. Helen Colley (University of Sheffield, UK; University Research Ethics Approval Reference Number 003463). Fibroblasts were maintained in Dulbecco’s Modified Eagle Medium (DMEM) supplemented with 10% Fetal Bovine Serum, L-glutamine and 100 U/ml penicillin and 100 μg/ml streptomycin. All materials were purchased from Sigma-Aldrich, USA. Fibroblasts were incubated in a humidified atmosphere containing 5% CO_2_ at 37 ^0^C. Cell passages from 1 to 7 were used in this study. For TGF-β1 treatment, fibroblasts were incubated with ranging concentrations of TGF-β1 (0.05–5 ng/ml) for 24 h and/or 48 h.

### Quantitative real-time PCR

Total RNA was extracted from fibroblasts using the RNeasy mini kit (Qiagen, Germany) according to the manufacturer’s instructions. cDNA conversion of 100 ng RNA was done using the High-Capacity cDNA reverse transcription kit (Life Technologies, UK) according to the manufacturer’s instructions. Total cDNA was amplified using the Power SYBR green PCR master mix (Life Technologies, UK). Primers sequence were as follows; U6 Forward (F) 5′CTCGCTTCGGCAGCACA3′, U6 Reverse (R) 5′AACGTTCACGAATTTGCGT3′, αSMA F 5′GAAGAAGAGGACAGCACTG3′, αSMA R 5′TCCCATTCCCACCATCAC3′, fibronectin-1 with extra domain A (FN-EDA1) 5′TGGAACCCAGTCCACAGCTATT3′, FN-EDA1 R 5′GTCTTCTCCTTGGGGGTCACC3′. Relative quantitation of targets in different samples was calculated by the ΔΔCt method, normalised to endogenous control U6.

### Immunocytochemistry

Fibroblasts were fixed with methanol (Fisher-Scientific, UK) for 20 min and permeabilised by using 4 mM sodium deoxycholate (Fisher-Scientific, UK) for 10 min. Cells were blocked for non-specific binding sites in 2.5% (w/v) bovine serum albumin in phosphate buffered saline (PBS) for 30 min, followed by incubating with a FITC-conjugated anti-human α-SMA mouse antibody (clone 1A.4; 1:100; Sigma-Aldrich,USA) for overnight at 4°C. Coverslips were washed twice with PBS, mounted using mounting media containing 4′,6-diamidino-2-phenylindole (DAPI; Vector Laboratories Inc, USA), viewed using a Ziess Axioplan 2 fluorescence light microscope at 40x magnification and photographed using Proplus 7.0.1 image software.

### Gel contraction assay

Fibroblasts (2.5 x 10^5^ cells/well) were mixed with 4 mg/ml of rat tail collagen (Roche,UK) in DMEM and pH adjusted to 7 using 0.1 mM NaOH (Sigma-Aldrich,UK). The cell:collagen mixture was added to 24 well plates and incubated for 45 min for gel to polymerize. The gels were incubated overnight at 37 ^0^C in serum-free DMEM. The gels were then loosened from the edges of the well by a scalpel and were incubated with serum free medium containing 5 ng/ml of TGF-β1 for 48 h. Collagen lattices were photographed and the surface area for each lattice was calculated using the Image J.

### Immunoblotting

Total protein lysates were prepared in radioimmunoprecipitation assay buffer (Sigma-Aldrich, USA) supplemented with complete mini protease inhibitor cocktail (Roche, UK). Protein quantification was determined by BCA protein assay kit (Thermo Scientific, UK). Protein lysates (20 μg) were resolved by 12% (v/v) sodium dodecyl sulphate-polyacrylamide gel electrophoresis (SDS-PAGE) and transferred onto nitrocellulose membrane (Milipore, USA) by iBlot dry transfer (Life technologies, UK). Following blocking of non-specific protein binding, membranes were incubated with mouse monoclonal anti-human αSMA (1:1000; Sigma–Aldrich USA) or mouse monoclonal β-actin (1:4000, Sigma-Aldrich, USA) at 4°C overnight. After a few washing steps, the membrane was incubated with a horseradish peroxidase-conjugated secondary antibody (1:3000, Cell Signalling Technology, USA) for 1 h at 37°C. All antibodies were diluted in 5% (w/v) skimmed milk powder and 3% (w/v) bovine serum albumin in Tris-buffered saline (TBS) with 0.05% (v/v) Tween 20 (TBS-T). The membrane was visualised by enhanced Pierce chemiluminescence (Thermo Scientific, UK). Densitometry was performed using Image J.

### Taqman Tiling Low-Density Array (TLDA)

miRNA expression profiling was performed using commercially available Tiling Low-Density Array; TLDA (Life Technologies, UK), according to the manufacturer’s protocol. Briefly, total RNA (3 ng) was used as an input for retrotranscription using a TaqMan MicroRNA Reverse Transcription Kit (Life Technologies, UK) and the TaqMan Megaplex pool kit (Life Technologies, UK), which are customised to run the TLDAs. Preamplification was performed using TaqMan PreAmp Master Mix (Life technologies, UK) according to the manufacturer’s protocol. RT-PCR was carried out using a 7900HT thermocycler (Applied Biosystems, USA). The raw data were analyzed by Data Assist Software version 3.0 (Life Technologies, UK). The ΔCt values were normalized to U6 endogenous control at a Ct cut-off value of 34, according to technical recommendation. EV-miRNA abundance was normalised to mean Ct value across all detected miRNA in each sample with a detection cut-off of Ct 32. Fold change of the differentially expressed miRNAs were calculated from ΔΔCt values relative to the untreated sample.

### Extracellular vesicle isolation and characterisation

EV were isolated as described in Peacock et al [12]. Briefly, NOF or eCAF were rinsed with PBS and incubated in serum-free DMEM for 24–72 h. Conditioned medium collected and centrifuged at 300 x *g* for 10 min, 2000 x *g* for 10 min and 10,000 x *g* for 30 min. The supernatant was reduced to 0.5 ml using a Vivaspin-20 (100 kDa molecular weight cut-off) column (GE Healthcare, Buckinghamshire, UK). EV were isolated by size exclusion chromatography using Sepharose CL-2B (GE Healthcare, Uppsala, Sweden) stacked in disposable Econo-Pac columns (Biorad, Watford, UK) and eluted in PBS. Where required, EVs were pelleted by ultracentrifugation at 100,000 x *g* for 1 h. Size profile and quantification of EV was performed by nanoparticle tracking analysis using a Zetaview instrument (Particle-Metrix) according to the manufacturer’s instructions.

### Bioinformatics analyses

Target genes of candidate miRNAs were predicted using online bioinformatic tools; TargetScan (http://www.targetscan.org/vert_71/), miRDB (http://www.mirdb.org/), and miRWalk (http://zmf.umm.uniheidelberg.de/apps/zmf/mirwalk2/index.html). Venn diagrams (http://bioinformatics.psb.ugent.be/webtools/Venn/) were used to obtain overlapping target genes from the three bioinformatic tools. GO enrichment analyses of biological process (BP), cellular compartment (CC), molecular function (MF) and Kyoto Encyclopedia of Genes and Genomes (KEGG) pathway were obtained using the DAVID (https://david.ncifcrf.gov/) bioinformatics tool [13]. For EV-associated KEGG pathway analysis, DIANA miR-Path v3 was used [14]. A false discovery rate (FDR) <0.05 was used as the cut-off criteria.

### Statistical analyses

Data are expressed as the mean ± standard error of the mean (sem) from three independent experiments, unless otherwise stated. Statistical analyses were made between two groups using a paired two-tailed Student’s *t*-test (Graph Pad Prism 7), as appropriate and indicated in figure legends. A value of *p* < 0.05 was considered statistically significant.

## Results

### TGF-β1 induces myofibroblast transdifferentiation

**Fig 1:**
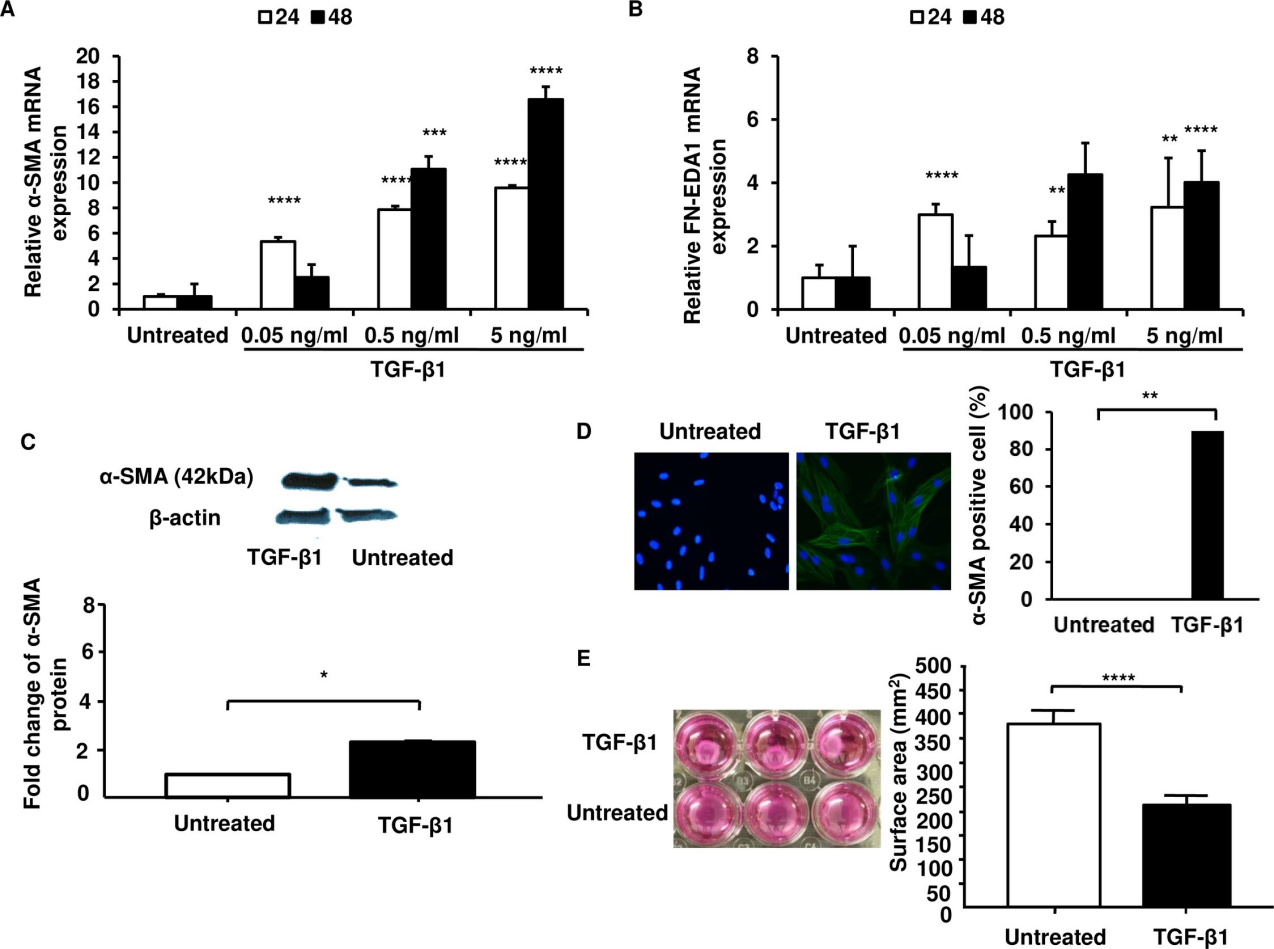
TGF-β1 induces myofibroblastic phenotype in human oral fibroblasts. Fibroblasts were treated with TGF-β1 (0.05–5 ng/ml) or serum-free medium (untreated), for 24 h and 48 h (A-B). Fibroblasts were subjected to RNA extraction, cDNA preparation and the expression of α-SMA (A) and FN-EDA1 (B) was determined using qPCR. Fibroblasts were treated with TGF-β1 (5 ng/ml) or serum-free medium (untreated) 48 h (C-E). Cell lysates were immunoblotted against α-SMA and β-actin (C). Fibroblasts were fixed in 100% methanol and were observed under a fluorescence microscope after being stained with the α-SMA-FITC antibody (green) and DAPI (blue) (D). The collagen gel contractility was assessed by measuring the gel surface area (E). Each data represents the mean ± SEM from three independent experiments. Statistical analysis was determined using two-tailed Student T-test with *p < 0.05, **p < 0.005, ***p < 0.001, ****p < 0.0001 compared to untreated.

In order to establish a model of CAF-like (eCAF) myofibroblast differentiation, we exposed NOF to TGF-β1. In keeping with previous reports, TGF-β1 induced expression of α-SMA and FN-EDA1 transcripts in eCAF with maximum α-SMA and FN-EDA1 expressions occurring at 48 h post-treatment at a concentration of 5 ng/ml (Fig 1A and 1B). Thus, this concentration and time point were used for subsequent experiments. Similarly, TGF-β1 caused increases in α-SMA protein abundance and the appearance of α- SMA-positive fibroblasts in eCAF (Fig 1C and 1D). The percentage of α- SMA positive cells in eCAF was significantly higher than that in NOF. These data are concordant with greater contractility of collagen 1 lattice in eCAF compared to NOF (Fig 1E).

### miRNAs are differentially expressed in eCAF

**Fig 2:**
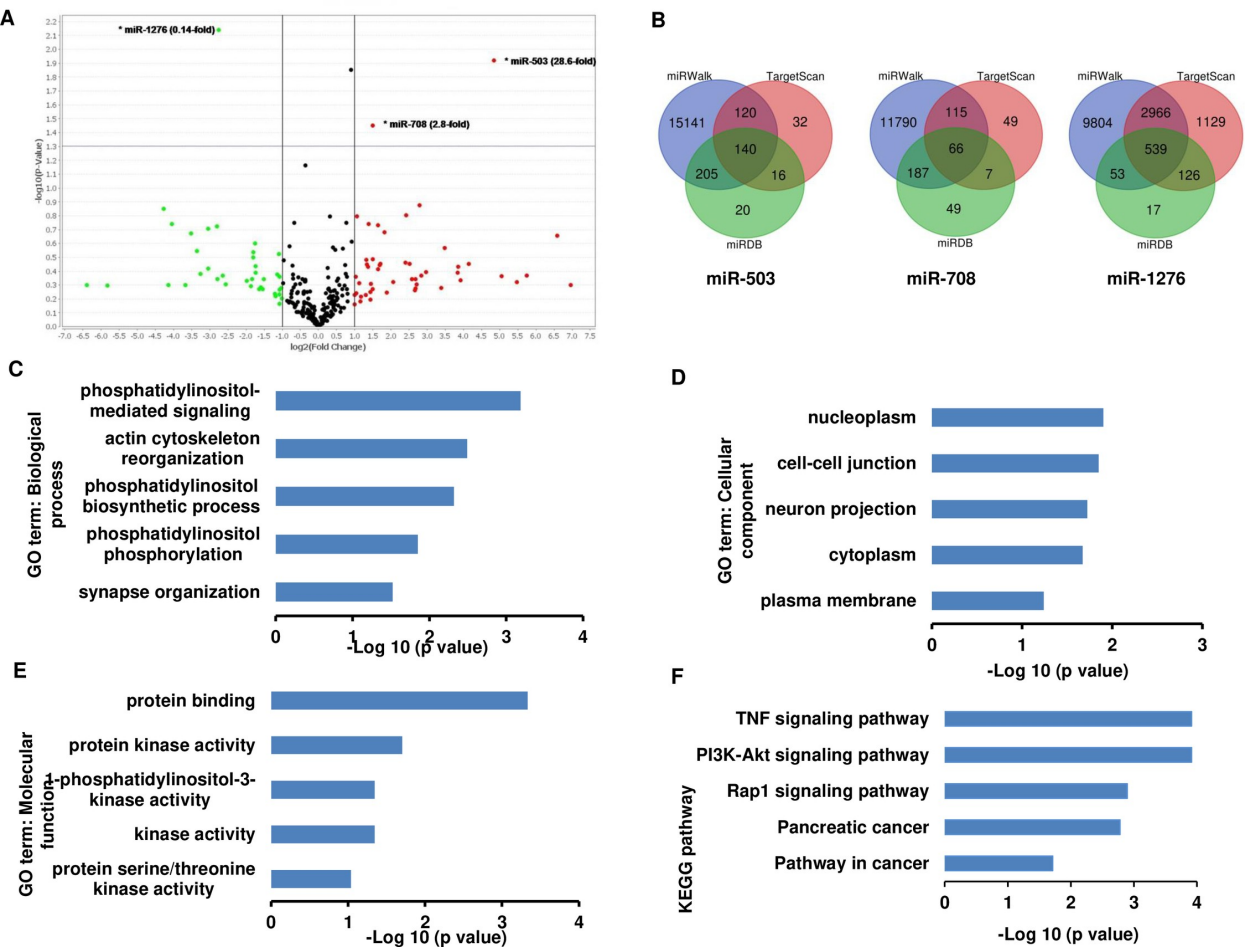
Differentially expressed miRNAs in myofibroblast transdifferentiation and their gene ontology and signaling pathway enrichment analysis. Fibroblasts were treated with or without TGF-β1 (5 ng/ml) for 48 h. RNA was extracted, cDNA was amplified and used for TLDA analysis. Only miRNAs above the horizontal line are significant (*p<0.05), the green dots denote miRNAs that are downregulated, red dots represent miRNAs that are upregulated, and the black dots are either miRNAs that are not significant or below the fold change cut off (A). Venn diagrams (http://bioinformatics.psb.ugent.be/webtools/Venn/) of target genes of each miRNAs using three bioinformatics tools (B). GO enrichment analysis demonstrated the top 5 genes enriched in biological process (BP) (C), cellular component (CC) (D), molecular function (MF) (E), and the KEGG pathway analysis (F).

It has been reported that several miRNAs are involved in myofibroblast transdifferentiation and alterations in their expression provoke or attenuate the CAF-like myofibroblastic phenotype. We next examined the expression of cellular miRNA in eCAF and NOF to gain insight to the potential role of miRNA in regulating the eCAF phenotype. We observed that only three miRNAs were differentially expressed in eCAF compared to NOF. Two miRNAs (miR-503; 28.6-fold and miR-708; 2.8-fold) were significantly upregulated, while miR-1276 was significantly downregulated (0.1-fold) in eCAFs (Fig 2A). In order to gain understanding of the potential biological significance of these changes, we used an *in silico* approach to identify potential target genes of miR-503, miR-708 and miR-1276 using three publicly available bioinformatics tools: TargetScan, miRDB, and miRWalk. As shown in Fig 2B, we identified a total number of 745 overlapping predicted target genes for these miRNAs from these algorithms. To understand the functional roles and mechanisms of identified target genes, GO and KEGG analyses were performed using DAVID. The results demonstrated that genes were enriched in five biological process (BP): synapse organization, phosphatidylinositol phosphorylation, phosphatidylinositol biosynthetic process, actin cytoskeleton reorganization and phosphatidylinositol-mediated signalling (Fig 2C). While for cellular component (CC), the genes were enriched in nucleoplasm, cell-cell junction, neuron projection, cytoplasm and plasma membrane (Fig 2D). Additionally, molecular function (MF) analysis showed that the genes were enriched in protein binding, protein kinase activity, 1-phosphatidylinositol-3-kinase activity, kinase activity, and protein serine/threonine kinase activity (Fig 2E). The KEGG pathway analysis indicated that the genes were mainly involved in pathways in cancer, Rap1 signalling pathway, PI3K-Akt signalling pathway, and TNF signalling pathway (Fig 2F).

### eCAF-derived extracellular vesicles display altered miRNA cargo

**Fig 3:**
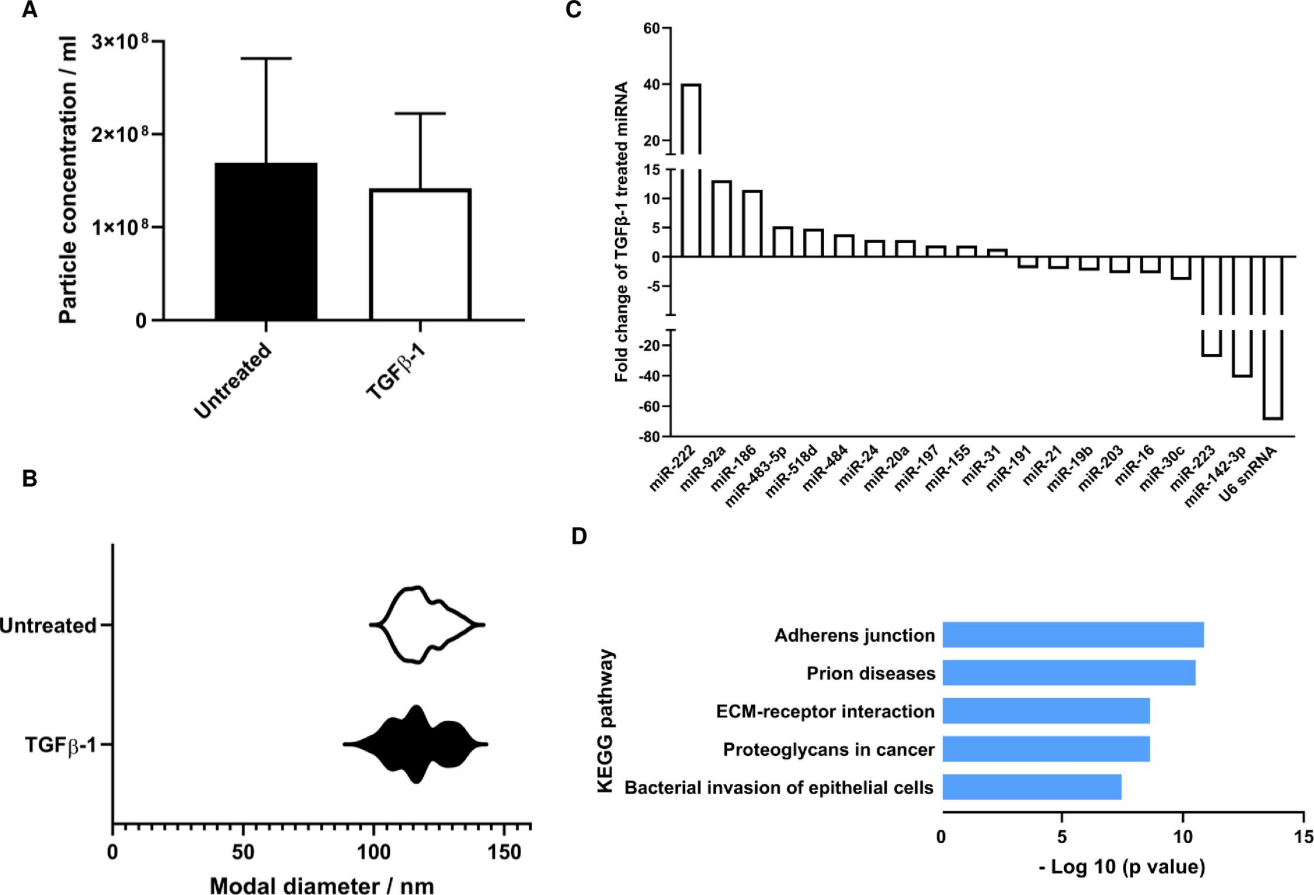
Characterisation of eCAF-derived EV and their miRNA cargo. EV were isolated from the conditioned media of fibroblasts treated with TGF-β1 (5 ng/ml) or their untreated counterparts using size-exclusion chromatography. The concentration (A) and size (B) of the isolated particles was analysed using nanoparticle tracking (Zetaview, Particle Metrix). Total RNA was extracted from isolated EV and their miRNA cargo was assessed using tiling low- density array quantitative PCR (C). KEGG pathway analysis on the three most elevated miRNA (miR-222, miR-92a and miR-186 combined) was conducted using DIANA (D) [14].

In recent years an understanding has emerged of the contribution of miRNA to intercellular signalling mediated by small membrane-enclosed moieties collectively termed EV [15]. The miRNA cargo of EV is reported to mediate numerous effects of cancer cell-derived EV in the tumour microenvironment, but considerably less is understood of the role EV-associated miRNA might play in CAF functionality. Having established changes in the cellular expression of miRNA in eCAF formation, we next therefore assessed the miRNA cargo of eCAF-derived EV compared to their normal fibroblast counterparts. We first isolated EV from NOF and eCAF and assessed their physical characteristics by nanoparticle tracking. No significant differences were observed in the size profile of NOF-EV compared to eCAF-EV, nor was there a significant difference between the number of EVs released by the different phenotypes (Fig 3A and 3B). miRNA profiling however revealed a number of differentially represented miRNA species (Fig 3C). Substantial differences in the abundance of a number of miRNAs were observed for EV isolated from eCAF compared to normal fibroblasts; this included a greater than ten-fold elevation in the abundance of miR-92a, miR-222 and miR-186, and a greater than ten-fold reduction in miR-223 and miR-142-3p (Fig 3C). Downregulation of the small nuclear RNA U6 negated its use as a reference gene for normalisation, in keeping with previous reports [16]. KEGG analysis of putative targets of the three most elevated miRNA in eCAF-EV (miR-222, miR-92a and miR-186) indicated that if delivered to recipient cells, the miRNA cargo has the potential to modulate pathways including ‘adherens junctions’, ‘extracellular matrix (ECM) receptor interaction’ and ‘proteoglycans in cancer’, key processes in the tumour microenvironment (Fig 3D).

## Discussion

Emerging evidence has demonstrated that miRNAs are involved in CAF activation from resident stromal fibroblasts. However, the heterogeneity of miRNA expression varies due to patient-to-patient variability. It is well-known that cancer cells secrete high levels of TGF-β, and this paracrine secretion is believed to trigger a transition of resident fibroblasts to CAF phenotype with myofibroblastic features in various cancers [17]. Therefore, with this in mind, the model for TGF-β-induced myofibroblast transdifferentiation was used to confirm that the phenotype assessed in this study were CAF-like-myofibroblastic cell. Myofibroblasts are commonly characterised by the expression of activation markers, α-SMA and *de novo* expression of fibronectin [18, 19]. Increased α-SMA and fibronectin expression is consistent with the study of Melling et al [3] and Dally et al [20]. α-SMA is rapidly incorporated into actin stress fibres, thereby increasing the contractility of fibroblasts [21]. The appearance of actin stress fibres is closely related to the generation of contractile force [22, 23]. In keeping with this, TGF-β1 provoked the development of α-SMA positive stress fibres and promoted the contractile phenotype of NOF. Herein, we show that TGF-β1 profoundly induces CAF-like-myofibroblastic phenotype in NOF.

miRNAs are likely to play a role in TGF-β1 signalling; most members of the TGF-β1 pathway are known or predicted to be targeted by one or more miRNAs [24]. Moreover, TGF-β1 directly regulates the biogenesis of miRNA through Smads [25]. We thus think that miRNA plays a role in myofibroblast transdifferentiation. We show that miR-503 and miR-708 were highly upregulated in eCAF, compared to NOF. Several studies demonstrated that miR-503 modulates myofibroblast transdifferentiation and fibrosis. Wu et al [26] demonstrated that miR-503 expression was decreased in TGF-β1 stimulated lung fibroblasts and upregulation of miR-503 attenuated CAF-like myofibroblastic phenotype in myofibroblasts. Several miRNAs are involved in cardiac fibrosis (reviewed in [27]), and another study has proposed that miR-503 modulates fibrosis by enhancing the ECM deposition in cardiac fibroblasts [28]. miR-708 is one of the more recently discovered miRNAs to play a role in fibrosis. A recent study demonstrated that miR-708 was upregulated in fibrotic liver tissues and ectopic expression of miR-708 inhibits hepatic stellate cell activation and reduces the ECM accumulation [29]. We also showed that miR-1276 was significantly downregulated in eCAF, compared to NOF. Several studies have shown that miR-1276 participates in cancer progression, however, to date, the functional role of miR-1276 in myofibroblast transdifferentiation or fibrosis has not been investigated. Taken together, these miRNAs might be involved in myofibroblast transdifferentiation. Further functional analyses using miRNA inhibitors or miRNA mimics will give a more definite understanding of the involvement of these miRNAs in myofibroblast transdifferentiation in the future.

GO analysis demonstrated that the deregulated genes were mainly involved in phosphatidylinositol-mediated signalling (ontology: BP), nucleoplasm (ontology: CC), and protein binding (ontology: MF). Previous study has revealed that phosphatidylinositol-mediated signalling regulates pro-inflammatory interleukin 32α (IL-32α) expression in human pancreatic periacinar myofibroblasts [30]. At the level of cellular component, the target genes were associated with miRNA translocation. Some miRNAs are present at much higher levels in the nucleolus and occur at very low levels in the nucleoplasm and/or cytoplasm [31]. As for the molecular function, numerous proteins including Smad7 have been reported to bind to TGF-β receptors [32]. The most enriched pathways from KEGG analysis were the TNF-signalling pathway and PI3K-Akt signalling pathway. TNF mediates various biological processes such as cell differentiation, cell proliferation, inflammation and apoptosis [33]. TGF-β can induce activation of PI3K-Akt indirectly via smad-dependent or–independent pathways [34, 35]. Additionally, TGF- β indirectly activates PI3K-Akt signalling by inducing the expression of several miRNAs. Previous studies have revealed that TGF- β activates the PI3K-Akt pathway by inducing the expression of miR-216a/217 and miR-21 in kidney cancer cells and hepatoma cells [36, 37].

Given the well-described roles for EV in modulating paracrine signalling in cancer and other contexts by the action of their miRNA cargo on target genes in recipient cells, it is potentially significant that the data presented here indicate the differential abundance of specific miRNA species in EV isolated from eCAF compared to NOF. Although requiring further validation, several of these miRNAs have known their putative roles in regulating the expression of a number of genes and pathways relevant to cancer. miR-92a, for example, the abundance of which was found to be increased by more than 10-fold in eCAF-derived EV, is widely reported to have pro-tumourigenic effects, with elevated abundance in EV isolated from cancer patients associated with poor survival [38]. The proposed mechanism of action of miR-92a is the promotion of epithelial-mesenchymal transition (EMT), a process favouring metastasis also known to be promoted by CAF-derived EV [38, 39]. Another miRNA identified here as elevated in eCAF-derived EV, miR-222, is also reported to promote EMT [40] and that elevated EV-associated miR-222 promotes tumourigenesis and correlates with poor survival in pancreatic cancer patients [41]. By contrast, miR-142-3p and miR-223, both found to be decreased in abundance in eCAF-derived EV, are widely ascribed tumour-suppressive functions [42, 43], although this may be context dependent. None of the miRNA found to be significantly up or down-regulated in the cellular transcriptome were altered in EV, which could imply selective loading of miRNA into EV, but it should be noted here that these samples weren’t obtained from the same culture used for cellular miRNA profiling and therefore differences between cultures cannot be excluded from accounting for this observation. Taken together, this provides preliminary evidence that the miRNA cargo of eCAF-derived EV has the potential to enhance tumour growth compared to that of EV isolated from NOF.

In summary, the findings reported here build on an emerging understanding of the role miRNA may play in the ability of TGF-β1 to induce a myofibroblastic CAF-like phenotype *in vitro*. The study also represents, to our knowledge, the first assessment of changes in EV miRNA cargo occurring during the transition of oral fibroblasts to a CAF-like myofibroblastic phenotype. Further experimental studies are needed to determine the functional role and the underlying mechanism (s) of these miRNAs.

## Supporting information

S1 Fig Uncropped and original Western blot against α-SMA from untreated and TGF-β1-treated NOF cells. Proteins were collected from 3 independent batches.
(TIF)
