# Supplementary figures and images for: Myofibroblast transdifferentiation is associated with changes in cellular and extracellular vesicle miRNA abundance
Source: PLoS One. 2021 Nov 11;16(11):e0256812. doi: 10.1371/journal.pone.0256812 (PMC8584782; doi:10.1371/journal.pone.0256812)

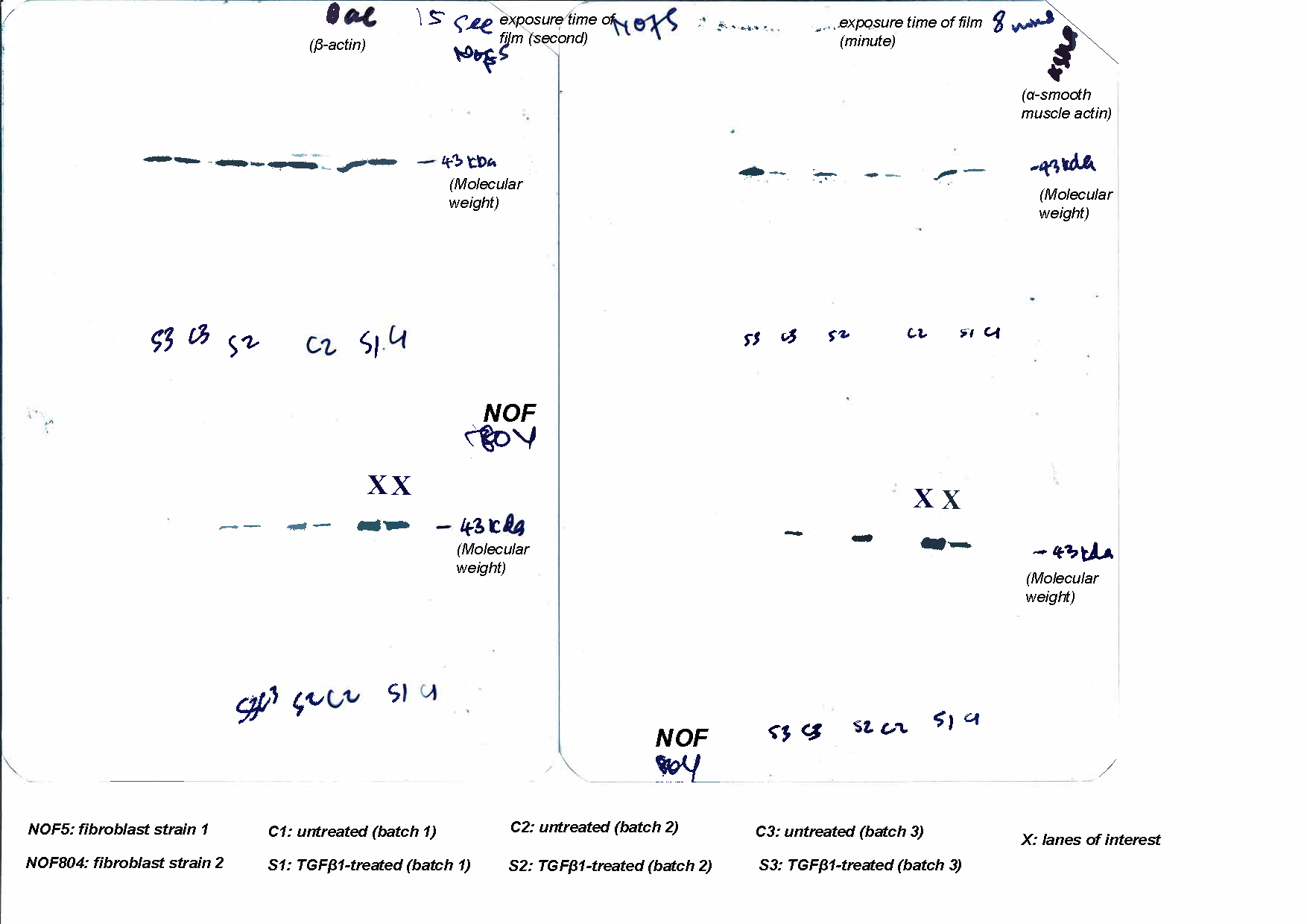

Supplement: S1 Fig — Proteins were collected from 3 independent batches. (TIF) [file pone.0256812.s001.tif]
